
JOURNAL INFORMATION
==============================
NLM Title Abbreviation: Inter Econ
Journal ID: Inter Econ
NLM Journal ID: 101086399

Inter Economics

ISSN: 0020-5346

ARTICLE INFORMATION
==============================
PMCID: PMC8664676
PMID: 34924599
DOI: 10.1007/s10272-021-1016-3
Article ID: 1016
Article version: 1
Subjects: Letter from America

America’s Polarized Politics of Climate Change

Furlow John j.furlow@iri.columbia.edu

Braun Mélody m.braun@iri.columbia.edu

grid.21729.3f 0000000419368729 International Research Institute for Climate and Society, Columbia University Climate School, 120 Monell, 61 Route 9W, 10964 Palisades, New York USA
Electronic publication date: 2021 Dec 11
Print publication date: 2021
Volume: 56
Issue: 6
First page: 371
Last page: 372
Copyright: © The Author(s) 2021
License: Open Access: This article is distributed under the terms of the Creative Commons Attribution 4.0 International License (https://creativecommons.org/licenses/by/4.0/).
Open Access funding provided by ZBW — Leibniz Information Centre for Economics.
License URL: https://creativecommons.org/licenses/by/4.0/

issue-copyright-statement: © The Author(s) 2021

==============================

Winston Churchill described the Soviet Union and its actions toward Germany at the beginning of WWII as "A riddle wrapped in a mystery inside an enigma." The same might be said of the United States' actions concerning climate change (among other issues). To understand whether Americans view the outcomes of the Glasgow Climate Change Conference as successes or failures, it is important to remember how divided the American population is politically. Every issue is rapidly politicized. Almost 800,000 people have now died of COVID-19 in America. In the early months of the pandemic, Republicans saw a political advantage in a slow response because the virus was mostly affecting states that were predominantly Democratic. Now, the anti-vaccine movement largely follows party lines: Maps of vaccination rates resemble voting patterns, with Biden-voting counties getting vaccinated and Trump-voting counties getting sick. This politicization pattern even made its way into support for the US at last summer's Olympics. Donald Trump proclaimed that, were it not for "leftist maniacs," the US Women's National Soccer team would have won the gold medal.

If politics can divide a country over taking steps to avoid unnecessary deaths or whether to support its own athletes in the Olympics, how can it unite on addressing climate change? This year's Conference of the Parties (COP) to the UN Framework Convention on Climate Change (UNFCCC) was the first conference since the US withdrew from, and then reentered, the Paris Agreement. The COVID-19 pandemic postponed COP26 from 2020 to 2021, preventing a meeting during the United States' brief self-imposed exile. Several US negotiators said how relieved they were that they did not have to attend a COP during the year when the US was out of Paris, but it was clear that Americans remained divided over the decision to rejoin. Interestingly, the issue has not always been so divisive. In 2008, the Republican Newt Gingrich and Democrat Nancy Pelosi pledged to join forces and fight climate change together. In 2007, 71% of Americans believed burning fossil fuels would affect the climate. This share fell to 51% in 2009, and 44% in 2011.

The US has had an enigmatic role in climate negotiations from the beginning. It provided about 40% of the total funding for the Intergovernmental Panel on Climate Change, the UN body that conducts scientific assessments to inform decision-making around climate. This funding was cut in 2017 by the Trump Administration. The first George Bush Administration negotiated the UNFCCC at the Rio Summit, and the US is a major funder of the UNFCCC secretariat. Under President Bill Clinton, US negotiators played a significant role in shaping the Kyoto Protocol, the 1997 treaty to curb greenhouse gas emissions through a trading scheme modeled on market-based approaches to limiting the pollutants that cause acid rain. The US never ratified Kyoto because the Senate unanimously voted for the 1997 Byrd-Hagel Resolution, which declared that the US should not join a climate agreement that would create new commitments for developed countries if it did not also create commitments for developing countries, or would harm the US economy.

This divided government attitude is partly a reflection of the different electoral calculations of a president who can draw votes from across the country, versus individual senators and congress members who are elected by voters in their states, or in even smaller congressional districts. The voting of members of congress does not seem to reflect national attitudes on climate. Just before COP26, a survey from the AP-NORC Center and the Energy Policy Institute at the University of Chicago showed that 75% of Americans believe climate change is happening, and 59% say it is accelerating. Fifty-five percent say they would pay a small surcharge on their electricity bills to fund more investment in renewable energy. While almost half the population would support requiring a transition to electric vehicles, the divide along political party lines helps explain why the polling does not translate into new laws: 61% of Democrats support such a requirement, while only 23% of Republicans do. The structure of our government means that America in 2021 is neither governed by averages nor, in some cases, by majorities.

So how will these attitudes make their way into practice? As with many issues in the US, the markets will drive action. US $CO_2$ emissions peaked in 2007 and have fallen by about 13% overall. Emissions from electricity generation have fallen by about one-third, due to a shift in fuel choice from coal to natural gas and renewables. As the cost of generating electricity from renewables and natural gas has fallen, electricity producers have shifted and cut emissions.

Emissions from transportation have only fallen slightly across the same period. The transportation sector is now the single biggest source of emissions in the country. To meet the Biden Administration's commitments to the world through the UNFCCC process, emissions from energy will have to continue to fall, and emissions from transportation will have to fall dramatically.

Americans are unlikely to give up their cars anytime soon, but the emissions from those cars can change. Teslas and other electric cars are increasingly popular. Cost and convenience are two factors that keep consumers from switching to electric cars, but the average retail cost of electric cars is expected to fall below that of gas-powered cars in the next few years. And the Democratic Party's second infrastructure bill, passed by the House of Representatives this November, contains a number of incentives and penalties to encourage a more rapid transition of the energy and transportation sectors including offering rebates for electric vehicle purchases of up to $12,000 per car and dramatically increasing the number of charging stations for electric cars. There are also tax credits for renewable energy production, investments in the electric grids so they can take in more power from renewables, as well as increased fees and penalties for fossil fuel extraction, use and pollution.

Americans lived through a summer of climate impacts in 2021, with drought and wildfires out west and storms and flooding in the east. The estimated cost of damages to property was close to $100 billion. The awareness that there are consequences for inaction is reflected in opinion surveys, in climate activism and in consumer behavior. Even Fox News seems to be shifting from denying climate change to arguing that it is good: several stories note that fewer people are dying from extreme cold. Will these changes in attitudes and behavior be enough for the United States to play the leadership role needed for the world to curb global emissions? Will economic carrots and sticks be enacted to allow American technological creativity to scale up solutions that the whole world could benefit from? That remains to be seen. The political divisions in the US pose formidable hurdles.

While progress at the national level is unsteady, we are seeing increasing commitments from the private sector and real dedication and leadership from cities and states. These efforts can support the actual implementation of the objectives of the Paris Agreement regardless of commitments of the federal government, while also acting as incentives to increase ambition at the federal level if included in the national effort. Another quote attributed to Winston Churchill offers some hope: "Americans can always be counted on to do the right thing, once they have exhausted all other possibilities."

John Furlow, International Research Institute for Climate and Society, Columbia University, Palisades, NY, USA

Mélody Braun, International Research Institute for Climate and Society, Columbia University, Palisades, NY, USA.

